# Evaluating vaccination dosing strategies for SARS-CoV-2 in patients at high-risk for allergic reactions: Insights from vaccination campaign^☆^

**DOI:** 10.1016/j.waojou.2025.101095

**Published:** 2025-07-28

**Authors:** Stefania Nicola, Iuliana Badiu, Nicolò Rashidy, Elena Saracco, Erika Montabone, Luca Lo Sardo, Marzia Boem, Valentina Marmora, Federica Corradi, Andrea Ricotti, Richard Borrelli, Giovanni Rolla, Simone Negrini, Luisa Brussino

**Affiliations:** a Department of Medical Sciences, University of Turin, Immunology and Allergy Unit, Mauriziano Hospital, 10128, Turin, Italy; b Hospital Administration Unit, Mauriziano Hospital, 10128, Turin, Italy

**Keywords:** COVID-19 vaccines, Vaccination, COVID-19, SARS-CoV-2, Pfizer-BioNTech COVID-19 vaccine, Moderna COVID-19 vaccine, ChAdOx1 nCoV-19 vaccine, Ad26COVS1 vaccine, NVX-CoV2373 vaccine, Hypersensitivity, Risk assessment, Polyethylene glycols, Polysorbates

## Abstract

**Background:**

The COVID-19 pandemic significantly increased the demand for allergy consultations to evaluate the risk of hypersensitivity reactions in patients either before receiving their first dose of an anti-SARS-CoV-2 vaccine (Group 1) or following suspected allergic reactions after vaccination (Group 2).

**Methods:**

We conducted a retrospective analysis of patients referred to the Immunology and Allergy Unit of the Azienda Ospedaliera Ordine Mauriziano in Turin, Italy, between December 2020 and December 2022. Risk assessment was performed according to Italian and European guidelines, and allergy skin tests were administered when necessary. Patient data were cross-referenced with the SIRVA platform (Regional Vaccination Management Information System) to assess vaccine eligibility, administration, and outcomes.

**Results:**

A total of 1222 patients were evaluated (mean age: 52 years; female-to-male ratio 4:1). In Group 1 (n = 914), 137 patients (15%) underwent skin testing, of whom 15 (1.6%) tested positive. Vaccination was recommended for 899 patients (98%), though 184 (20%) did not proceed. Among those vaccinated, 679 (74%) received additional doses, with 48% receiving a third and 11% a fourth dose. In Group 2 (n = 308), 104 patients (33%) underwent skin testing, with 9 (8%) testing positive. Vaccination without restrictions was recommended for 299 patients (97%), but 45 patients (15%) did not proceed. Among the remaining, 262 (85%) received a second dose, 183 (59%) a third, and 29 (9%) a fourth dose. Overall, 1198 patients (98%) had no specific contraindications to vaccination. Only 5 patients (0.4%) were completely exempted from vaccination due to confirmed sensitivity to both polyethylene glycol (PEG) and polysorbate 80 (PS80). An alternative vaccine was recommended for 19 patients; 16 of them proceeded with vaccination and tolerated it without adverse effects.

**Conclusions:**

Our findings demonstrate that the majority of high-risk allergic patients can safely receive anti-SARS-CoV-2 vaccines following allergological evaluation. The rate of confirmed excipient allergy was very low, and vaccine adherence was comparable to the general population. This is, to our knowledge, the first study to longitudinally assess the number and types of vaccine doses administered to high-risk allergic individuals.

## Introduction

Since COVID-19 was declared a pandemic in March 2020, vaccines against SARS-CoV-2 infection have been considered the most effective tool to combat the spread of the disease.^1^

The COVID-19 vaccination campaign began in Italy and across Europe on December 27, 2020. To date, 155.3 million doses have been administered in Italy, providing complete vaccination coverage to 50.8 million people, which corresponds to approximately 86.27% of the Italian population.^2^

There are several vaccines available against COVID-19 in our country: Pfizer-BioNTech, Moderna, both containing polyethylene glycols (PEGs) of a molecular weight of 2000 Da (PEG 2000), AstraZeneca, Janssen, and Novavax, all having polysorbate 80 Da (PS80) as their excipient. Trometamol was also used in the Moderna vaccine and lately in some Pfizer updated vaccine formulations. This excipient is also present in some NSAID, MRI, and CT scan contrast media and is potentially capable of causing anaphylaxis, as previously described in the literature for ketorolac.^3^

PEG and PS80 are commonly used as excipients in many medications, vaccines, and cosmetic products, and they could be the hidden culprits of many allergic reactions to those products.

Table 1 describes the COVID-19 vaccines currently on the market in Italy and their potential allergens.

A full vaccination course typically involves 2 doses for the Pfizer-BioNTech, Moderna, AstraZeneca, and Novavax, while Janssen only requires 1 dose.

An additional dose of mRNA COVID-19 vaccine may be considered for adults and adolescents aged 12 years and older with clinically relevant immunocompromise, such as solid organ transplant recipients. This additional dose can be administered at least 28 days after the last dose of the primary vaccination cycle and can be from 1 of the 2 mRNA vaccines authorized in Italy (Comirnaty and Spikevax).

For individuals contracting SARS-CoV-2 infection after the first dose of a two-dose vaccine regimen, the following guidance is provided:

- If the infection is confirmed within 14 days of receiving the first vaccine dose, completing the vaccine schedule with a second dose is recommended within 6 months from the documented infection date.
- If the infection is confirmed beyond the 14th day after the first vaccine dose, the vaccine schedule is considered complete as the infection itself is equivalent to receiving the second dose.

The booster doses are recommended for individuals of 60 years and older, healthcare professionals, and individuals with high vulnerability due to pre-existing pathologies aged 18 years and older. Booster doses can be administered at least 4 months after completing the primary vaccination cycle or the last event using 1 of the 2 mRNA vaccines authorized in Italy (Comirnaty or Spikevax). Heterologous vaccination (using a different mRNA vaccine type for the booster dose) is also allowed.^4^

In Italy since January 7, 2022, it has been mandatory for individuals over 50 years old to have completed a SARS-CoV-2 vaccination cycle, whereas for healthcare workers, this requirement has been in place since April 2021.^5^

Across all types of vaccines, the most frequently reported adverse effects include symptoms such as fever, fatigue, headaches, muscle and joint discomfort, injection site pain, chills, and nausea. Most of these events were categorized as non-severe and resolved by reporting time. Comparing after-first- and second-dose adverse event reporting rates with after-third-dose rates, the last ones consistently decreased, with 21.7 reported events per 100,000 administered doses.^6^

Regarding severe hypersensitivity reactions, cases of anaphylaxis associated with the SARS-CoV-2 vaccine, and its excipients have been reported since the beginning of the vaccination campaign.^6^, ^7^, ^8^

In accordance with Italian and European guidelines, a previous anaphylactic reaction to the vaccine or a positive result in skin tests for PEG or PS80 in a patient with a suggestive history of allergy to medications containing PEG or PS80, contraindicate the administration of a vaccine containing that same excipient. However, vaccination with an alternative vaccine is possible.^9^^,^^10^

The previous exposure to the vaccine excipients through other medications could sensitize the immune system, explaining why some patients may experience an allergic reaction event with the first dose of the vaccine. Therefore, it is mandatory to recommend avoidance of all medications and cosmetic products containing those excipients in patients identified with a certified sensitization to PEG and PS80.^11^

Similarly, the quadrivalent human papillomavirus (HPV) vaccine (Gardasil) contains PS80, which was already found to cause hypersensitivity reactions in 2012 due to its excipient. However, the list of medications and vaccines containing PS80 and PEG is very long and should be carefully analyzed by physicians with expertise in this field when taking the history of patients with previous allergic reactions.

Despite the number of papers in the literature describing the safety of a second or third vaccine dose in patients who underwent careful allergy evaluation, data regarding the overall number of doses and the types of vaccines administered remain lacking.^12^, ^13^, ^14^, ^15^, ^16^, ^17^, ^18^

This is the first paper that aims to longitudinally describe the types and quantity of anti-SARS-CoV-2 vaccine doses administered in patients evaluated as being at high risk of allergic reactions developing allergic reactions to specific vaccination (Group 1) and in patients with suspected IgE-mediated reactions to the COVID-19 vaccine (Group 2).

Therefore, this study aims to provide an overview of how the vaccination campaign in Piedmont has progressed and its outcome in patients assessed for high-risk allergic conditions. Our results highlight how, even in high-risk specific populations, vaccination adherence was similar to that of the general population after allergological work-up.2 2.

## Materials and methods

### Study design

**Fig. 1:**
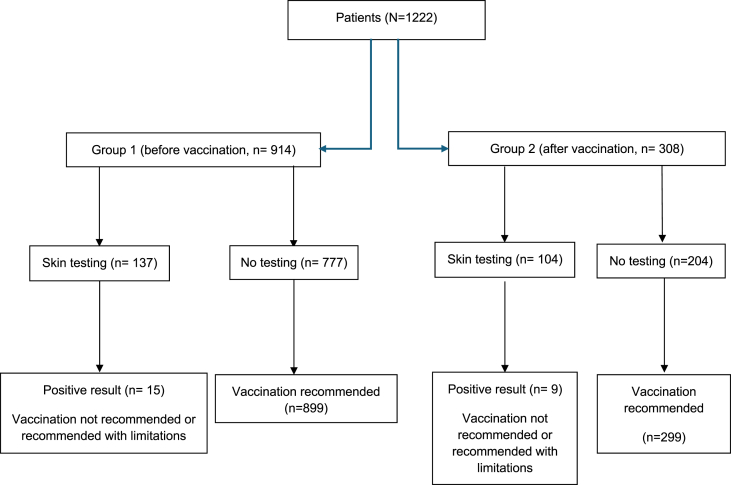
Study flowchart

**Fig. 2:**
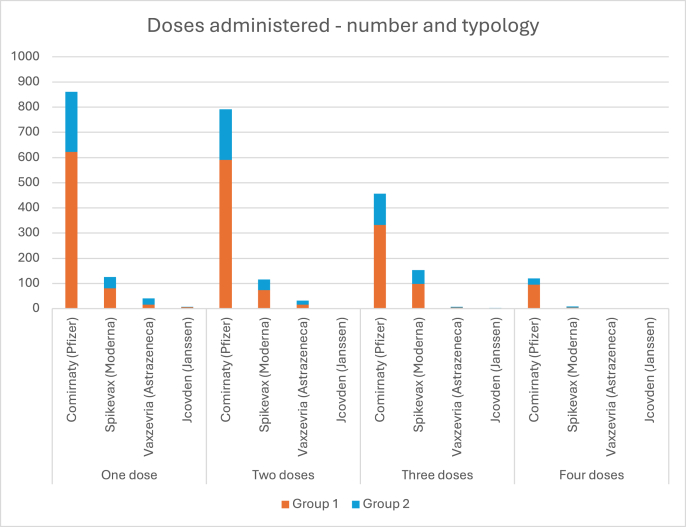
Distribution of vaccine administration in high-risk allergic patients. The y-axis represents the number of patients, while the x-axis represents the type of vaccine administered

A retrospective analysis was conducted on patients who underwent evaluation for COVID-19 vaccination at the Allergy and Immunology Unit of Mauriziano Hospital in Turin, Italy between December 2020 and March 2023. The study was conducted in compliance with the Declaration of Helsinki and Good Clinical Practice guidelines, under the Italian decree-law No. 18 of March 17, 2020, article 17-bis on the faculty of processing health-related data during the state of emergency for purposes of substantial public interest in the field of public health. Study diagram can be seen in Fig. 1 (see Fig. 2).

### Patients

We analyzed all outpatients evaluated at our Unit either prior to COVID-19 vaccination—based on potential risk—or following suspected hypersensitivity reactions to vaccine excipients (PEG/PS80) or to specific COVID-19 vaccines.

Patients already undergoing treatment for systemic mastocytosis, asthma, anaphylaxis, or other allergic conditions were excluded from the study.

Two distinct groups were identified: Group 1 included unvaccinated individuals considered at high risk for hypersensitivity reactions to COVID-19 vaccines, while Group 2 comprised patients who had received at least 1 vaccine dose and subsequently developed suspected hypersensitivity reactions.

Patients were stratified into low- and moderate-to-high-risk categories based on their medical history (both self-reported and from clinical assessments by other healthcare professionals), the internal guidelines of Mauriziano Hospital, the recommendations of the Italian Societies for Allergology, Asthma, and Clinical Immunology (AAIITO/SIAAIC), and the European Academy of Allergy and Clinical Immunology (EAACI).^9^^,^^10^

Specifically, low-risk patients were defined as individuals with allergic reactions to foods, inhalants, latex, or medications not containing PEG or PS80. Moderate-to-high-risk patients included those with a suggestive history of hypersensitivity to drugs containing PEG and/or PS80, or those who experienced immediate hypersensitivity reactions (within 4 h) following a prior COVID-19 vaccine dose. Delayed reactions were defined if observed within 14 days post-vaccination.

Allergy testing was performed in high-risk patients who had experienced acute reactions to PEG- and/or PS80-containing drugs, provided no other PEG/PS80-containing medications had been administered. Patients with probable acute hypersensitivity reactions to anti-SARS-CoV-2 vaccines were also evaluated.

According to national and European guidelines, skin testing was not required in all cases. Patients with a low-risk clinical history were considered eligible for vaccination without undergoing allergological testing. As a result, the number of patients for whom vaccination was recommended exceeds the number who underwent skin testing.

Additional data were collected, including the number of patients undergoing skin testing for vaccine excipients, their vaccination eligibility, recommended post-vaccination observation periods, use of premedication therapy, and the need for administration in a monitored hospital setting.

The Regional Information System for Vaccine Management (SIRVA) was used to cross-reference allergy assessment data with the patients’ subsequent vaccination history. Data on the type of vaccine administered (Comirnaty, Spikevax, Vaxzevria, or Janssen) and the number of doses received were analyzed.

### Statystical analyses

Statistical analysis was performed using Statistica 10.0 (StatSoft Inc., Tulsa, OK, USA). For categorical variables, rates and proportions were calculated, while medians and ranges were used for continuous variables. The Wilk–Shapiro test was applied to assess normality. As the data did not follow a normal distribution, the two-tailed Mann–Whitney *U* test was used to compare continuous variables, while the Chi-square (χ^2^) test was employed for categorical comparisons. A p-value of <0.05 was considered statistically significant. To focus the analysis on actual vaccine tolerance in high-risk patients and to avoid variability introduced by different premedication regimens, we chose not to include premedication as a variable.

## Results

A total of 1222 patients were evaluated; demographics are presented in Table 1.

**Table 1:** Summary of COVID-19 vaccines available in Italy and their potential allergens. This table lists the vaccines Comirnaty (Pfizer-BioNTech), Spikevax (Moderna), Vaxzevria (AstraZeneca), Johnson & Johnson (Janssen), and Nuvaxovid (Novavax) along with their respective potential allergens, such as polyethylene glycol (PEG 2000), trometamol, and polysorbate 80 (PS80)

Vaccines	Potential allergens
Cominarty (Pfizer-BioNTech)	Polyethylene glycol (PEG 2000)
Spikevax (Moderna)	Polyethylene glycol (PEG 2000), trometamol
Vaxzevria (AstraZeneca)	Polysorbate (PS80)
Johnson & Johnson	Polysorbate (PS80)
Nuvaxovid (Novavax)	Polysorbate (PS80)

Among the total number of patients evaluated and divided into 2 groups, we reported the number who underwent allergy testing, the number diagnosed with allergies, and the type of exemption (total or partial) recommended. We also recorded the number of patients who received the first, second, third, and fourth vaccine doses, the type of vaccine administered, and the number of patients who, despite a favorable recommendation from the allergy specialist, did not proceed with vaccination.

Overall, after the allergy evaluation, 81% of patients (988 individuals) proceeded with vaccination, while 19% (229 individuals) chose not to receive the vaccine. The CONSORT (CONsolidated Standards of Reporting Trials) chart can be seen in Fig. 1.

Table 2 presents the 2 groups of patients with undergoing skin tests, positive skin tests and limitations to vaccination.

**Table 2:** Characteristics of patients undergoing skin tests, including test results and limitations to vaccination. This table compares 2 groups of high-risk patients evaluated for suspected hypersensitivity reactions

	Group 1	Group 2	Total
Total evaluated patients - n°	914	308	1222
Age (y ± SD)	53 ± 16	47 ± 18	52
Female sex - n° (%)	722 (79%)	262 (85%)	984 (80%)
Patients undergoing skin testing - n° (%)	137 (15%)	104 (33%)	241 (20%)
Positive skin test - n° (%)	15 (1,6%)	9 (3%)	24 (2%)
Complete exemption from vaccination - n° (%)	4 (0,4%)	1 (0,3%)	5 (0,4%)
Partially exempted from vaccination - n° (%)	11 (1,2%)	8 (2,7%)	19 (1,6%)
Patients without limitations to vaccinationv	899 (98,4%)	299 (97%)	1198 (98%)
Patients who did not undergo vaccination after specialist assessment - n° (%)	184 (20%)	45 (15%)	229(19%)
Mean age - yr	52 ± 15	45 ± 15	52
Female sex - n° (%)	148 (16%)	34 (11%)	182 (15%)
Patients who underwent vaccination after specialist assessment – n° (%)	726 (80%)	262 (85%)	988 (81%)

Table 3 presents the number and typology of anti-SARS-CoV-2 vaccines administered in high-risk patients.

**Table 3:** Number and types of anti-SARS-CoV-2 vaccines administered in high-risk allergic patients. This table details how many doses of each vaccine were administered to the study population

Doses administered - n° (%)	Group 1 (n = 914)	Group 2 (n = 308)	Total (n = 1222)
**One dose**	**726 (80%)**	**308**	**1034 (85%)**
Comirnaty (Pfizer)	623 (86%)	238 (77%)	861 (83,3%)
Spikevax (Moderna)	81 (11%)	45 (14,7%)	126 (12,2%)
Vaxzevria (Astrazeneca)	16 (2%)	24 (8%)	40 (3,8%)
Jcovden (Janssen)	6^a^ (1%)	1^a^ (0,3%)	7 (0,7%)
**Two doses**	**679 (74%)**	**262 (85%)**	**941 (77%)**
Comirnaty (Pfizer)	590 (87%)	201 (76,8%)	791 (84%)
Spikevax (Moderna)	73 (11%)	43 (16,4%)	116 (12,4%)
Vaxzevria (Astrazeneca)	16 (2%)	16 (6%)	31 (3,4%)
Jcovden (Janssen)	0	2 (0,8%)	2 (0,2%)
**Three doses**	**436 (48%)**	**183 (59%)**	**619 (51%)**
Comirnaty (Pfizer)	332 (76%)	124 (67,8%)	456 (73,6%)
Spikevax (Moderna)	98 (22,5%)	55 (30%)	153 (24,7%)
Vaxzevria (Astrazeneca)	4 (1%)	3 (1,6%)	7 (1,2%)
Jcovden (Janssen)	2 (0,5%)	1 (0,6%)	3 (0,5%)
**Four doses**	**100 (11%)**	**29 (9%)**	**129 (11%)**
Comirnaty (Pfizer)	95 (95%)	25 (86%)	120 (93%)
Spikevax (Moderna)	5 (5%)	4 (14%)	9 (7%)
Vaxzevria (Astrazeneca)	0	0	0
Jcovden (Janssen)	0	0	0

a Jannsen vaccine required a single dose to complete the vaccination cycle.

### Group 1

Among the 914 patients (average age 53 ± 16 years; 722 females) evaluated before vaccination due to suspected allergies to excipients, 137 (15%) underwent skin testing. Of these, 15 patients (1.6%) tested positive and were diagnosed with an excipient allergy.

Following the allergy evaluation, 899 patients (98%) were recommended for vaccination without any restrictions.

Of the 15 patients allergic to excipients, 4 were completely exempted from all vaccines due to a cross-allergy between PEG and polysorbate. Six patients were exempted only from vaccines containing PEG, as they tested negative for PS80, while 5 patients were exempted only from PS80-containing vaccines, having tested negative for PEG.

All 11 patients with partial exemptions followed our recommendations and received the alternative vaccine. None required revaluation after vaccination.

Table 4 presents the results of skin tests and vaccine exemptions for 15 patients who have undergone evaluation after experiencing systemic reactions to drugs containing PEG or PS80.

**Table 4:** Results of skin tests and vaccine exemptions in Group 1. This table presents data on patients who underwent skin testing for vaccine excipients and those who were granted exemptions from specific vaccines

Pre-Vaccinal Evaluation Group (Group 1)															
Patient n°	Sex	Age	Systemic reactions		Skin test				Complete Exemption	Partial Exemption				Vaccines administered after evaluation	
			Drugs containing PEG	Drugs containing PS80	Depomedrol (PEG3350)	PEG 6000/4000	Kenacort (Polisorbato 80)	Comirnaty (Pfizer)		Comirnaty	Spikevax	Vaxzevria	Jcovden	N° doses	Type
1	F	22	•	•	**POS +**	**POS +**	**POS +**	**POS +**	•					None	
2	F	40	•	•	**POS +**	**POS +**	**POS +**	N.E.	•					None	
3	F	51	•		**POS +**	**POS +**	**POS +**	NEG -	•					None	
4	F	55	•		NEG -	**POS +**	NEG -	N.E.		•	•			1	Jcovden (Janssen)
5	F	19	•		**POS +**	N.E.	**POS +**	N.E.	•					None	
6	F	53	•	•	**POS +**	NEG -	NEG -	N.E.		•	•			2	Vaxzevria (Astrazeneca)
7	F	31		•	NEG -	N.E.	**POS +**	N.E.				•	•	None	
8	F	55	•	•	NEG -	N.E.	**POS +**	N.E.				•	•	3	Spikevax (Moderna)
9	M	71	•	•	**POS +**	**POS +**	NEG -	N.E.		•	•			2	Vaxzevria (Astrazeneca)
10	F	61	•		**POS +**	N.E.	NEG -	N.E.		•	•			1	Jcovden (Janssen)
11	M	57	•		N.E.	**POS +**	NEG -	N.E.		•	•			2	Vaxzevria (Astrazeneca)
12	M	73		•	NEG -	N.E.	**POS +**	N.E.				•	•	4	Comirnaty (Pfizer)
13	F	48	•		**POS +**	N.E.	NEG -	N.E.		•	•			2	Vaxzevria (Astrazeneca)
14	F	38		•	NEG -	N.E.	**POS +**	N.E.				•	•	3	Comirnaty (Pfizer)
15	F	29	•	•	NEG -	N.E.	**POS +**	N.E.				•	•	3	Comirnaty (Pfizer)

Reviewing the data from the SIRVA platform, we found that 20% of the evaluated patients, totaling 184 individuals (148 women, average age 52 years), did not receive any vaccination despite a positive recommendation from the specialist. Among these 184 patients, 110 (63%) had never had an allergy consultation before; for 80% (140 patients), it was their first allergy visit related to drug allergies.

Of the remaining 80% (722 patients) who received the SARS-CoV-2 vaccination (Group 1), 679 (94% of those who had the first dose) went on to receive a subsequent dose (second or third, depending on the vaccine used). Additionally, 436 patients (64%) received an additional dose, and 100 patients (11%) received 4 doses.

The most administered vaccine across all doses was Pfizer, accounting for 86% of the first and second doses, 76% of the third, and 95% of the fourth dose. Moderna followed, while AstraZeneca and Johnson & Johnson were used in a very small percentage of patients.

### Group 2

Of the 308 patients evaluated for a suspected allergic reaction to the vaccine (238 Pfizer, 45 Moderna, 24 AstraZeneca, 1 Johnson & Johnson), 307 experienced reactions after the first dose, while only 1 patient had a reaction after the second dose (Pfizer vaccine).

A total of 115 patients (39%) had reactions compatible with an allergy, including 29 cases despite antihistamine premedication. Among them, 57 patients had immediate reactions: 27 experienced isolated itching, 28 developed acute urticaria, 34 had respiratory distress, 2 experienced transient hypotension of short duration that resolved without medical intervention (possibly related to vasovagal syndrome), and 6 had both urticaria and respiratory distress without hypotension, which are classified as anaphylaxis according to the Brighton Collaboration criteria.^19^^,^^20^

Fifty-three patients experienced delayed reactions, presenting as urticaria (45 patients), isolated itching (23 patients), or maculopapular rash (33 patients), with no severe reactions reported. Additionally, 7 patients experienced an exacerbation of asthma, 8 had a worsening of chronic spontaneous urticaria, and 3 had a flare-up of an underlying autoimmune disease (1 early arthritis, 1 psoriatic arthritis, and 1 erythema nodosum in a patient with sarcoidosis).

Table 5 describes the types of adverse reactions to SARS-CoV-2 vaccination.

**Table 5:** Types of adverse reactions to SARS-CoV-2 vaccination. This table categorizes the different hypersensitivity reactions observed in the study, including immediate and delayed reactions

	Patients - No. (%)
Group 2	308
Immediate reactions	132 (43%)
Respiratory distress	42 (14%)
Urticaria	28 (9%)
Isolated itching	27 (9%)
Urticaria and respiratory symptoms	6 (2%)
Transient hypotension	2 (0,6%)
Delayed reactions	158 (51,3%)
Maculopapular rash	67 (22%)
Urticaria	50 (16%)
Isolated itching	23 (7%)
Asthma exacerbation	7 (2,2%)
Chronic Sponaneous urticaria exacerbation	8 (2,6%)
Exacerbation of underlying autoimmune pathology (1 early-arthritis, 1 PsA, 1 erythema nodosum in a patient with sarcoidosis)	3 (1%)

Of the 308 patients, 104 (33%) underwent skin testing, and of these, 9 (8%) tested positive, confirming allergic hypersensitivity to the vaccine excipients. One patient with an immediate cutaneous reaction to the Pfizer vaccine tested positive for both PEG and PS80 and was completely exempted from vaccination. Eight patients received partial exemptions: 1 allergic only to PS80 and 7 allergic only to PEG.

Table 6 summarizes the results of skin tests and vaccine exemptions for these 9 patients who experienced allergic reactions to SARS-CoV-2 vaccination.

**Table 6:** Results of skin tests and vaccine exemptions for patients in Group 2. This table provides data on patients who experienced suspected allergic reactions after the first vaccine dose and the results of their subsequent allergological assessments

Post-Vaccine Reaction Group (Group 2)															
Patient n°	Sex	Age	Previous vaccine	Previous adverse reaction	Skin test				Complete Exemption	Partial Exemption				Vaccines administered after evaluation	
					D epomedrol (PEG3350)	PEG 6000/4000	Kenacort (Polisorbato 80)	Comirnaty (Pfizer)		Comirnaty	Spikevax	Vaxzevria	Jcovden	N° doses	Type
1	F	50	COMIRNATY (Pfizer)	Immediate urticaria	N.E.	**POS +**	**POS +**	**POS +**	•					None	
2	M	19	COMIRNATY (Pfizer)	Immediate urticaria	**POS +**	N.E.	NEG -	N.E.		•	•			1	Vaxzevria (Astrazeneca)
3	F	41	COMIRNATY (Pfizer)	Immediate asthma exacerbation	**+/−**	**POS +**	N.E.	**POS +**		•	•			None	
4	F	38	COMIRNATY (Pfizer)	Immediate urticaria	**POS +**	NEG -	NEG -	NEG -		•	•			1	Vaxzevria (Astrazeneca)
5	F	45	VAXZEVRIA (Astrazeneca)	Immediate urticaria	NEG -	N.E.	**POS +**	N.E.				•	•	2	Comirnaty (Pfizer)
6	F	31	COMIRNATY (Pfizer)	delayed urticaria	+/−	N.E.	NEG -	**POS +**		•	•			1	Jcovden (Janssen)
7	F	41	COMIRNATY (Pfizer)	delayed urticaria	**POS +**	N.E.	NEG -	N.E.		•	•			1	Jcovden (Janssen)
8	F	51	COMIRNATY (Pfizer)	Immediate urticaria	**POS +**	N.E.	N.E.	N.E.		•	•			1	Vaxzevria (Astrazeneca)
9	F	44	COMIRNATY (Pfizer)	Immediate urticaria	POS +	N.E.	NEG -	**POS +**		•	•			None	

Six patients were subsequently vaccinated with an alternative vaccine: 2 received Johnson, 3 received AstraZeneca, and 1 received Pfizer. Two patients did not receive further vaccination.

Following allergy evaluation, 299 patients (97%) were deemed eligible for vaccination without limitations. However, 15% of patients in Group 2 (n = 45) did not proceed with vaccination. A second dose of SARS-CoV-2 vaccine was administered to 262 patients (85%), a third dose to 183 (59%), and a fourth dose to 29 patients (9%).

As in Group 1, Pfizer was the most commonly administered vaccine across all doses (77% for the first, 76% for the second, 68% for the third, and 86% for the fourth dose), followed by Moderna, AstraZeneca, and Johnson.

Table 7 and Fig. 1 provide further details on the type and number of vaccines administered.

**Table 7:** Number and types of vaccine doses administered in Group 1 and Group 2. This table summarizes the vaccination history of high-risk allergic patients, including first, second, third, and fourth doses

Doses administered - n° (%)	Group 1	Group 2	Total
**One dose**	**726 (80%)**	**308**	**1034 (85%)**
Comirnaty (Pfizer)	623 (86%)	238 (77%)	861 (83,3%)
Spikevax (Moderna)	81 (11%)	45 (14,7%)	126 (12,2%)
Vaxzevria (Astrazeneca)	16 (2%)	24 (8%)	40 (3,8%)
Jcovden (Janssen)	6∗ (1%)	1∗ (0,3%)	7 (0,7%)
**Two doses**	**679 (74%)**	**262 (85%)**	**941 (77%)**
Comirnaty (Pfizer)	590 (87%)	201 (76,8%)	791 (84%)
Spikevax (Moderna)	73 (11%)	43 (16,4%)	116 (12,4%)
Vaxzevria (Astrazeneca)	16 (2%)	16 (6%)	31 (3,4%)
Jcovden (Janssen)	0	2 (0,8%)	2 (0,2%)
**Three doses**	**436 (48%)**	**183 (59%)**	**619 (51%)**
Comirnaty (Pfizer)	332 (76%)	124 (67,8%)	456 (73,6%)
Spikevax (Moderna)	98 (22,5%)	55 (30%)	153 (24,7%)
Vaxzevria (Astrazeneca)	4 (1%)	3 (1,6%)	7 (1,2%)
Jcovden (Janssen)	2 (0,5%)	1 (0,6%)	3 (0,5%)
**Four doses**	**100 (11%)**	**29 (9%)**	**129 (11%)**
Comirnaty (Pfizer)	95 (95%)	25 (86%)	120 (93%)
Spikevax (Moderna)	5 (5%)	4 (14%)	9 (7%)
Vaxzevria (Astrazeneca)	0	0	0
Jcovden (Janssen)	0	0	0

### Overall analysis

Out of the 1222 patients, only 5 were completely exempted from vaccination (4 in Group 1 and 1 in Group 2), 19 received partial exemption, 16 proceeded with alternative vaccination, and no patient returned for the revaluation.

Vaccination was recommended in almost all patients (1198 patients, 98%). After the allergy evaluation total of 988 patients (81%) followed the specialist's recommendation and received almost 1 dose, but 233 patients (19%) did not proceed with vaccination. No statistical difference was found in comparing Group 1 and Group 2 regarding adherence to the first vaccination after specialist evaluation. We further analyzed the introduction of the vaccination mandate (January 7, 2022), but we did not find any statistically significant difference in adherence to the received indication (p: 0.8).

No patient in Group 1 nor in Group 2 was revaluated after vaccination for further adverse reactions.

## Discussion

During the COVID-19 vaccination campaign, there was a substantial increase in allergy consultations due to uncertainty about vaccine safety, particularly in individuals with suspected allergies to specific excipients or to the vaccine itself. This situation required significant efforts to implement structured allergy assessments conducted by specialists, aimed at reducing hypersensitivity reactions (HSRs), ensuring accurate diagnoses, and enabling the successful vaccination of nearly all patients.

It is worth noting that the vaccination rate in our study population (81%) was slightly lower than the national average (86.27%). This difference may be partly explained by the high-risk profile of our cohort, in which vaccine hesitancy may have persisted despite specialist evaluation and recommendation. Psychological factors, such as fear of allergic reactions, especially in patients undergoing their first allergological consultation, may have played a role in the decision not to proceed with vaccination.

While several studies have outlined the management of high-risk allergic patients during the SARS-CoV-2 vaccination campaign, data on the number of doses and the types of vaccines administered following allergy evaluation remain limited.^21^^,^^22^

HSRs to COVID-19 vaccines or their excipients are rare.^23^ In our study, 1.6% (15 out of 914) of patients considered at high risk of an hypersensitivity reaction (HSR) prior to vaccination were sensitized to a vaccine excipient, compared to 8% (9 out of 308) of patients who had experienced an HSR to a previous COVID-19 vaccine dose. Two clinical studies reported similarly low sensitization rates: Petrelli et al found a 3% positivity rate (5 out of 152), with 3 patients in the high-risk pre-vaccination group and 2 in the post-vaccine reaction group.^24^ Similarly, Montera et al reported a 0.02% positivity rate (10 out of 362) among patients with a history of PEG/PS80 drug allergy, and 7.1% (12 out of 169) among those with prior vaccine reactions.^25^

Conversely, a recent Italian study reported a higher sensitization rate of 12.7% (16 out of 126), likely attributable to a highly selected patient population with an elevated pre-test probability.^26^

Interestingly, all these studies found higher positivity rates among patients tested after experiencing vaccine reactions, compared to those tested based on prior history alone. However, the predictive value of allergy tests for PEG and PS80 remains uncertain, as not all sensitized patients develop reactions upon vaccination.^15^^,^^25^

In our cohort, 24 patients tested positive for vaccine excipients. Among them, 5 patients (4 from Group 1 and 1 from Group 2) were granted a complete exemption from vaccination due to confirmed sensitization to both PEG and PS80. The remaining 19 patients were sensitized to only 1 excipient, which enabled the administration of an alternative vaccine, in accordance with Italian and European guidelines.^9^^,^^10^ Of these, 16 (84%) were successfully vaccinated without adverse reactions, while 3 (16%) chose not to proceed. These results underscore the feasibility of safe vaccination even in patients with confirmed excipient allergies. Nevertheless, current data on the safety of alternative vaccines in this population remain limited.^23^, ^24^, ^25^

Immunologically, it has been hypothesized that PEG and PS80 may act as haptens, binding to proteins and forming complexes capable of triggering an immune response. Despite their structural differences, both excipients may share immunogenic epitopes, leading to cross-reactivity via IgE recognition of short PEG motifs.^27^ Moreover, PEGylated drugs have been shown to induce anti-PEG immunoglobulins, which may contribute to anaphylactic reactions to anti-SARS-CoV-2 vaccines.^28^

In Group 2, 9 out of 308 patients (3%) tested positive for excipient allergies via skin testing. These findings are consistent with previous reports indicating sensitization rates of 2.8%–7.1% among patients with a history of PEG or PS80 hypersensitivity.^24^^,^^25^ This group exhibited a spectrum of HSRs, ranging from mild symptoms (eg, pruritus, urticaria) to more severe manifestations such as anaphylaxis—primarily characterized by concurrent urticaria and dyspnea, with no cases of severe hypotension.

Overall, 98% of patients were deemed eligible for vaccination following allergological evaluation, with only 0.4% receiving a complete exemption. Post-evaluation, 81% (988 individuals) adhered to vaccination recommendations, while 19% (229 individuals) declined. This adherence rate aligns with general population trends, suggesting that comprehensive allergological assessments and tailored guidance can help address concerns and promote vaccination among high-risk individuals.^2^^,^^29^^,^^30^

Despite initial concerns, 77% of patients received at least 2 doses, and 50% of the entire study population proceeded with additional booster doses. These findings highlight the pivotal role of allergy specialists in managing complex cases and facilitating safe, broad vaccine coverage—even within high-risk populations.

In light of recent consensus recommendations, the role of allergy testing prior to vaccination has been further refined. The consensus suggests that in the majority of cases, routine excipient skin testing with PEG or polysorbate 80 may have limited diagnostic value and should not delay vaccination unnecessarily. Our data align with this approach, showing that most high-risk patients tolerated vaccination well; this supports the emerging view that a detailed clinical history should guide the risk stratification process, reserving skin testing for select cases with strong suggestive histories.

### Limitations

Our data do not offer insight into the complete vaccination cycle, including 2 doses or 1 dose plus infection, as we cannot track intervening infectious events or vaccinations outside our region. Additionally, the SIRVA platform lacks a dedicated string for reporting adverse reactions, relying instead on optional notes. However, vaccinating physicians referred patients with suspected allergic hypersensitivity to our Center through a preferential booking pathway.

The lack of increased adherence following the vaccination mandate may have been affected by not knowing all the patient professions, as healthcare workers, teachers, and law enforcement personnel were already subject to the mandate by the middle of 2021.

We excluded premedication as a variable to maintain clarity in analyzing vaccine tolerance in high-risk patients. The presence of premedicated patients (n = 27) in group 2 suggests that some mild potential vaccine reactions might have been masked by antihistamine therapy.

## Conclusions

This study sheds light on how patients with high-risk allergic reactions to COVID-19 vaccines adhere to vaccination and their related outcomes.

This is the first study specifically designed to describe the number and types of doses administered to patients with high allergological risk, and we also demonstrate that their adherence was comparable to that of the general population. Only a minor portion of patients was exempt or partially exempt from vaccination, indicating the overall safety and manageability of the vaccine process for high-risk individuals. Future vaccination campaigns can rely on these insights to boost confidence and compliance among high-risk groups, enhancing overall vaccination rates and public health.

## Abbreviations

SARS-CoV-2, Severe Acute Respiratory Syndrome Coronavirus 2; COVID-19, Coronavirus Disease 2019; PEG, Polyethylene Glycol; PS80, Polysorbate 80; NSAID, Non-Steroidal Anti-Inflammatory Drug; MRI, Magnetic Resonance Imaging; CT, Computed Tomography; SIRVA, Regional Vaccination Management Information System; EAACI, European Association for Allergy and Clinical Immunology; AIITO, Italian Association of Allergology, Asthma, and Clinical Immunology; SIAAIC, Italian Society of Allergology, Asthma, and Clinical Immunology; HSR, Hypersensitivity Reaction; HPV, Human Papillomavirus; mRNA, Messenger Ribonucleic Acid; X², Chi-Square Test; SD, Standard Deviation; CI, Confidence Interval; OR, Odds Ratio; WHO, World Health Organization; EMA, European Medicines Agency; FDA, Food and Drug Administration; IgE, Immunoglobulin E; Da, Dalton; HCP, Healthcare Professional

## Declaration of Consent to Publish

All the authors confirm their consent for publication.

## Availability of Data and Materials

The data and materials will be made available upon request.

## Ethics Statement

In response to the request for clarification, our Hospital was allowed to proceed and use clinical data without requiring explicit informed consent during the COVID-19 pandemic in Italy under the Decree-Law No. 18 of March 17, 2020. Specifically, Article 17-bis of this decree established that personal data, including health-related data, could be processed during the state of emergency for purposes of substantial public interest in the field of public health. The provision aimed to facilitate the implementation of necessary health protection measures and the effective management of the national health emergency. This exceptional regulatory framework allowed the use of health data under the condition that such processing complied with the principles and requirements outlined in the General Data Protection Regulation (EU Regulation 2016/679) (GDPR) and the Italian Data Protection Code (Legislative Decree No. 196/2003).Importantly, the decree provided for specific exemptions regarding the requirement of individual informed consent when research was conducted on anonymized or pseudonymized datasets, ensuring that such activities remained compliant with applicable privacy and data protection laws.

## Author’s Contribution

S.N., I.B., L.B. conceived and designed the study. Data collection was performed by S.N., I.B., N.R., E.S., E.M., L.L.S., M.B., V.M., F.C., and R.B. Statistical analysis was conducted by A.R. The original manuscript draft was written by I.B., R.B., E.M., and M.B. The final manuscript revision and editing were carried out by I.B. and S.N. All authors read and approved the final manuscript.

## Funding

This paper was not funded.

## Declaration of competing interest

The authors declare no COI.

## References

[1] Liu Y., Li D., Han J. (2024). COVID-19 vaccines and beyond. Cell Mol Immunol.

[2] https://ourworldindata.org/explorers/covid?zoomToSelection=true&facet=none&uniformYAxis=0&country=∼ITA&pickerSort=asc&pickerMetric=location&hideControls=false&Metric=Vaccine+doses&Interval=7-day+rolling+average&Relative+to+population=true.

[3] Scala E., Giani M., Pirrotta L. (2001). Selective severe anaphylactic reaction due to ketorolac tromethamine without nonsteroidal anti-inflammatory drug intolerance. J Allergy Clin Immunol.

[4] https://www.aifa.gov.it/vaccini-covid-19.

[5] (2022).

[6] Risma K.A., Edwards K.M., Hummell D.S. (2021). Potential mechanisms of anaphylaxis to COVID-19 mRNA vaccines. J Allergy Clin Immunol.

[7] Sellaturay P., Nasser S., Islam S., Gurugama P., Ewan P.W. (2021). Polyethylene glycol (PEG) is a cause of anaphylaxis to the Pfizer/BioNTech mRNA COVID-19 vaccine. Clin Exp Allergy.

[8] McSweeney M.D., Mohan M., Commins S.P., Lai S.K. (2021). Anaphylaxis to Pfizer/BioNTech mRNA COVID-19 vaccine in a patient with clinically confirmed PEG allergy. Front Allergy.

[9] (2022). Linee Di Indirizzo per L’Inquadramento E La Gestione Dei Pazienti a Rischio Di Reazioni Allergiche Ai Vaccini per Il COVID-19.

[10] Sokolowska M., Eiwegger T., Ollert M. (2021). EAACI statement on the diagnosis, management and prevention of severe allergic reactions to COVID-19 vaccines. Allergy.

[11] Caballero M.L., Krantz M.S., Quirce S., Phillips E.J., Stone C.A. (2021). Hidden dangers: recognizing excipients as potential causes of drug and vaccine hypersensitivity reactions. J Allergy Clin Immunol Pract.

[12] Chu D.K., Abrams E.M., Golden D.B.K. (2022). Risk of second allergic reaction to SARS-CoV-2 vaccines: a systematic review and meta-analysis. JAMA Intern Med.

[13] Patel G.B., Chhiba K.D., Chen M.M. (2021). COVID-19 vaccine-related presumed allergic reactions and second dose administration by using a two-step graded protocol. Allergy Asthma Proc.

[14] Lim X.R., Tan J.W.L., Chan G.Y.L. (2022). Evaluation of patients with vaccine allergies prior to mRNA-Based COVID-19 vaccination. Vaccines.

[15] Wolfson A.R., Robinson L.B., Li L. (2021). First-dose mRNA COVID-19 vaccine allergic reactions: limited role for excipient skin testing. J Allergy Clin Immunol Pract.

[16] Stehlin F., Mahdi-Aljedani R., Canton L. (2022). Intradermal testing with COVID-19 mRNA vaccines predicts tolerance. Front Allergy.

[17] Tunbridge M., Perkins G., Lee M. (2022). COVID vaccination can be performed in patients with a history of allergic reactions to the vaccines or their components: experience from a specialist clinic in South Australia. Intern Med J.

[18] Rasmussen T.H., Mortz C.G., Georgsen T.K. (2021). Patients with suspected allergic reactions to COVID-19 vaccines can be safely revaccinated after diagnostic work-up. Clin Transl Allergy.

[19] Gold M.S., Amarasinghe A., Greenhawt M. (2023). Anaphylaxis: revision of the brighton collaboration case definition. Vaccine.

[20] Nicola S., Lo Sardo L., Borrelli R. (2024). Beyond the appearances: exploring complexities in anaphylaxis differential diagnosis. Curr Opin Allergy Clin Immunol.

[21] Messina M.R., Crisciotti C., Pellegrini L. (2023). Desensitization protocols for Anti-SARS-CoV-2 vaccines in patients with high risk of allergic reactions. Vaccines.

[22] Alhumaid S., Al Mutair A., Al Alawi Z. (2021). Anaphylactic and nonanaphylactic reactions to SARS-CoV-2 vaccines: a systematic review and meta-analysis. Allergy Asthma Clin Immunol.

[23] Paoletti G., Pepys J., Bragato M. (2023). The prevalence of immediate hypersensitivity reactions to the BNT162b2 mRNA vaccine against SARS-CoV-2: data from the vaccination campaign in a large academic hospital. Vaccines.

[24] Petrelli F., Giannini D., Pucci C. (2023). Allergy workup in the diagnosis of COVID-19 vaccines-induced hypersensitivity reactions and its impact on vaccination. Int Arch Allergy Immunol.

[25] Montera M.C., Giordano A., Asperti C. (2024). The role of skin tests with polyethylene glycol and polysorbate 80 in the vaccination campaign for COVID-19: results from an Italian multicenter survey. Eur Ann Allergy Clin Immunol.

[26] Nappi E., Racca F., Piona A. (2023). Polyethylene glycol and polysorbate 80 skin tests in the context of an allergic risk assessment for hypersensitivity reactions to Anti-SARS-CoV-2 mRNA vaccines. Vaccines.

[27] Ieven T., Coorevits L., Vandebotermet M. (2023). Endotyping of IgE-Mediated polyethylene glycol and/or polysorbate 80 allergy. J Allergy Clin Immunol Pract.

[28] Crisafulli S., Cutroneo P.M., Luxi N. (2023). Is PEGylation of drugs associated with hypersensitivity reactions? An analysis of the Italian national spontaneous adverse drug reaction reporting system. Drug Saf.

[29] https://www.governo.it/it/cscovid19/report-vaccini/.

[30] Badiu I., Nicola S., Rashidy N. (2024). How a novel approach of allergy call center improved the management of the Anti-COVID vaccination campaign in Piedmont: italy. J Epidemiol Glob Health.

